# Draft Genome Sequence of *Corynebacterium ulcerans* FRC58, Isolated from the Bronchitic Aspiration of a Patient in France

**DOI:** 10.1128/genomeA.01132-13

**Published:** 2014-01-09

**Authors:** Andréia do Socorro de Sousa Silva, Rafael Azevedo Baraúna, Pablo Caracciolo Gomes de Sá, Diego Assis das Graças, Adriana Ribeiro Carneiro, Maxime Thouvenin, Vasco Azevedo, Edgar Badell, Nicole Guiso, Artur Luiz da Costa da Silva, Rommel Thiago Jucá Ramos

**Affiliations:** Institute of Biological Sciences, Federal University of Pará (Universidade Federal do Pará—UFPA), Belém, Pará, Brazila; Hospital Center of Troyes, Microbiology Department, Troyes, Franceb; Institute of Biological Sciences, Federal University of Minas Gerais (Universidade Federal de Minas Gerais—UFMG), Belo Horizonte, Minas Gerais, Brazilc; Institut Pasteur, Unité Prévention et Thérapies Moléculaires des Maladies Humaines, Centre National de Référence des Corynebactéries du Complexe *diphtheriae*, Paris, Franced; CNRS-URA 3012, Paris, Francee

## Abstract

*Corynebacterium ulcerans* is a bacterial species with high importance because it causes infections in animals and, rarely, in humans. Its virulence mechanisms remain unclear. The current study describes the draft genome of *C. ulcerans* FRC58, which was isolated from the bronchitic aspiration of a patient in France.

## GENOME ANNOUNCEMENT

*Corynebacterium ulcerans* is a toxigenic, catalase-positive, nitrate-negative bacterial species that belongs to the CMNR (*Corynebacterium*, *Mycobacterium*, *Nocardia*, and *Rhodococcus*) group. Analyses of 16S rRNA have revealed that within this group, *C. ulcerans* is most closely related to the species *Corynebacterium pseudotuberculosis* and *Corynebacterium diphtheriae* (1). *C. ulcerans* can produce various clinical pictures in both humans and domesticated or wild animals. This bacterial species is an emerging pathogen that has been isolated from infectious conditions in various countries, including Brazil (2), Japan (3), Germany (4), England (5), and France (6). The frequently observed signs and symptoms of *C. ulcerans* infection are similar to those of classical diphtheria. This similarity occurs because *C. ulcerans* carries genes encoding phospholipase D (PLD) and diphtheria toxin (DT), which are regarded as the major virulence factors produced by *C. pseudotuberculosis* and *C. diphtheriae*, respectively (5, 7, 8). In certain lineages, such as *C. ulcerans* 809, which was isolated from a woman with a fatal lung infection in Rio de Janeiro, Brazil, the sequences of these virulence factors can vary greatly from the descriptions of the *Corynebacterium* genes provided in the literature (2). These differences can explain the lack of classical diphtheria symptoms in certain cases of *C. ulcerans* infection. Even in the absence of DT production, *C. ulcerans* can cause lower respiratory tract infections; moreover, similarly to the pathogenicity of nontoxigenic *C. diphtheriae* strains, the pathogenicity of *C. ulcerans* does not depend on the production of DT (4, 9, 10). This scenario demonstrates the need to obtain genomic data for the emergent pathogen *C. ulcerans* to describe the virulence mechanisms of this bacterial species. In fact, little knowledge is available regarding the *C. ulcerans* virulence factors associated with infectious conditions (11) and the pathogen-host interaction process. To date, only three genomes for this species have been entered into the National Center for Biotechnology Information (NCBI) database (7, 8). Two of these lineages were considered to be nontoxigenic because they lack the DT gene.

The FRC58 lineage of *C. ulcerans* examined in the present study was isolated from the secretions of an 86-year-old patient with bronchitis who was hospitalized at the Hospital Center of Troyes (France). The genome of this bacterial species was sequenced using the Ion Torrent PGM system, using a fragment library. The sequencing process generated 6,686,040 reads (~2 gigabases), which represents a coverage of 800×.

The reads were assembled *de novo* using the CLC Genomics Workbench. This assembly produced a total of 241 contigs with an N_50_ contig length of 113 kb; the longest contig is 419 kb, and the shortest contig is 201 bp. The contigs were annotated using Rapid Annotations using Subsystems Technology (12), and 2,503 coding sequences (CDSs), 10 rRNAs, and 61 tRNAs were identified. The G+C content of the examined genome is 53.23%. The complete *C. ulcerans* FRC58 genome was obtained, with a total of 2,609,412 bp.

### Nucleotide sequence accession numbers.

The *C. ulcerans* FRC58 draft genome sequence has been deposited in GenBank under the accession no. AYTI00000000. The version described in this paper is version AYTI01000000.
